# Adapting monocular SLAM for surface-trace free 3D registration in navigated arthroscopy

**DOI:** 10.1007/s11548-026-03631-1

**Published:** 2026-04-30

**Authors:** Tânia Baptista, Carolina Raposo, Miguel Marques, Diogo Vaz, Michel Antunes, Joao P. Barreto

**Affiliations:** 1 Institute of Systems and Robotics, University of Coimbra, Coimbra, Portugal; 2 Smith&Nephew, Coimbra, Portugal

**Keywords:** Surgical navigation, Monocular SLAM, Contactless 3D registration, Arthroscopy

## Abstract

**Purpose:**

Video-based surgical navigation (VBSN) relies on registering a preoperative 3D model to intraoperative data to overlay the surgical plan onto the patient’s anatomy, providing real-time guidance. In current systems, registration is achieved by tracking two visual markers: one rigidly attached to the anatomy, called the anatomy marker, and another mounted on a digitizing tool. The surgeon manually collects 3D points by contacting the anatomical surface with the tool tip. This workflow requires anatomy and tool markers to be simultaneously visible and continuous tool-tissue contact, which is time-consuming and may pose risks to the patient. In an attempt to overcome these difficulties, this paper investigates the possibility of removing visual markers from VBSN.

**Methods:**

Recent monocular Simultaneous Localization and Mapping (SLAM) approaches are adapted to the arthroscopic context to estimate both the camera pose and a 3D reconstruction of the scene from arthroscopic video.

**Results:**

Experimental results indicate that the anatomy marker cannot be removed, as camera pose estimation using SLAM does not simultaneously achieve the accuracy and real-time performance required for surgical navigation. However, the digitizing tool can be eliminated, enabling contactless registration using a SLAM-based variant. Moreover, SLAM can recover both structure and motion in short time segments without the anatomy marker, allowing the surgeon to inspect regions of the anatomy without constantly maintaining the anatomy marker in the field of view.

**Conclusion:**

The proposed SLAM-based approach advances VBSN by enabling real-time, contactless registration compatible with clinical requirements, as validated in cadaveric arthroscopy experiments with registration accomplished on the fly.

**Supplementary Information:**

The online version contains supplementary material available at 10.1007/s11548-026-03631-1.

## Introduction

Arthroscopy is a minimally invasive procedure used to treat joint pathologies, in which an arthroscopic camera and surgical instruments are introduced into the joint through small incisions. Although it reduces surgical trauma compared to open surgery, arthroscopy remains challenging due to indirect visualization of the anatomy and the limited maneuverability of instruments within the joint. Prior studies have shown that arthroscopy can benefit from surgical navigation [1, 2], where the surgical plan, usually defined on a preoperative model of the targeted tissue or bone, is overlaid with the patient anatomy in the OR through a 3D registration process [3, 4], and surgical execution is guided by tracking and determining the 6D pose of instruments with respect to said anatomy. The tracking can be accomplished by different means or sensing modalities including infra-red (IR) cameras [5], electromagnetic coil [6], and video. This article will focus on video-based surgical navigation (VBSN) that is specially well suited for arthroscopy because it can leverage the already existing video to accomplish computer navigation.

**Fig. 1:**
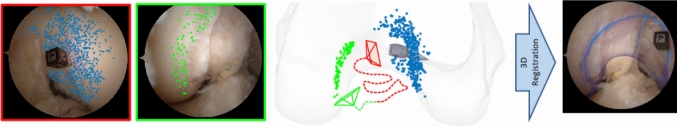
Arthro-vSLAM+, a hybrid vSLAM system, reconstructs the anatomical surface using marker-based camera poses when the anatomy marker is visible (red) and the Arthro-vSLAM module, based on CudaSIFT-SLAM, when the marker is not visible (green). The 3D map is initialized only when the anatomy marker is visible, and vSLAM camera pose optimization is disabled when poses are provided by the anatomy marker tracker. A 3D registration step [4] aligns the reconstructed points (green and blue) with the preoperative model, enabling preoperative information to be shown in augmented reality (blue overlay in right image)

VBSN, first introduced in [1], uses the intraoperative arthroscopic video to track visual markers rigidly attached to the target anatomy, henceforth referred to as the anatomy marker, as well as a digitizing tool and other instruments. The anatomy marker and digitizing tool are used to reconstruct 3D points, which are then fed to a registration algorithm [4] that aligns the preoperative 3D model with the anatomy. The current VBSN system [1] has demonstrated high registration accuracy in the context of anterior cruciate ligament (ACL) reconstruction. However, it presents two main limitations: (a) the requirement to screw an anatomy marker onto the bone to compute the arthroscopic camera pose, which must be seen at all times to determine camera poses and accomplish navigation, and (b) the need to acquire 3D points by touching, or contacting, the joint surface with a digitizing tool, a time-consuming process during surgery that requires skill and that, in specific situations, might introduce risk of iatrogenic damage due to the need of contact with delicate anatomical structures such as cartilage. The goal of this paper is to address limitations (a) and (b). An illustration of these limitations is provided in Fig. 1 of the Supplementary Material.

An appealing strategy to address the aforementioned limitations is to employ visual Simultaneous Localization and Mapping (vSLAM) [7]. In this context, limitation (i), which concerns the need to estimate the arthroscopic camera pose, can be overcome through the localization component of vSLAM, whereas limitation (ii), concerning the acquisition of 3D points, can be addressed by its mapping component where reconstructed structures are fed into a registration algorithm. In this paper, we thoroughly evaluate state-of-the-art monocular vSLAM pipelines in the context of VBSN for ACL reconstruction, assessing whether their localization and mapping accuracy meet surgical requirements, and whether their runtime performance satisfies real-time guidance demands in the operating room (minimum of 15 fps).

In summary, the main contributions of the paper are:

1. The introduction of Arthro-vSLAM and Arthro-vSLAM+. The former is a specialization to arthroscopic scenes of the CudaSIFT-SLAM pipeline that proved to be more effective than ORB-SLAM in endoscopic applications [8], and the latter further leverages the presence of the anatomy marker to accurately determine the camera pose and accelerate point matching whenever the marker is in the field of view (FOV).
2. Experimental tests of Arthro-vSLAM in footage with accurate ground truth for camera pose and structure that was acquired in 7 ex vivo experiments in ACL surgery using VBSN. The tests evaluate both camera motion recovery (localization) and structure estimation (mapping) without relying in the anatomy fiducial. Although the results obtained with Arthro-vSLAM are encouraging, clearly outperforming other vSLAM pipelines reported in literature as being applicable to arthroscopy [9], it is shown that accuracy and robustness of camera pose estimation are insufficient to safely guide power tools during surgery.
3. Considering the need of maintaining a fiducial marker attached to anatomy, it is demonstrated that Arthro-vSLAM+ is effective in accomplishing contactless bone 3D registration using only visual information, achieving accuracy comparable to conventional VBSN where a tracked touch probe is used to obtain the 3D structure. This performance is enable by the fact that in Arthro-vSLAM+ camera pose estimation can be accomplished in footage segments where the marker is out of sight ultimately leading to reconstruction of broader areas with positive impact in final registration (refer to Fig. 1 and Video 1 in the Supplementary Material).

## Background and related work

### Review of SLAM frameworks

The literature on SLAM is vast, offering several approaches that could serve as plausible solutions for the application described herein. Some works are specific for arthroscopy [10, 11], but they typically rely on sensors other than the arthroscopic camera, namely an external camera and the odometry of a robotic arm. Another family of SLAM pipelines works with video RGB-D as they consider a depth channel as input [12–14]. Since we intend to rely on the existing video provided by the arthroscopic camera, we will focus our attention only in visual SLAM (vSLAM).

There exist several deep learning-based vSLAM approaches in the literature that have proven to work well in indoor and outdoor scenarios. However, some of them are not a viable solution for VBSN in arthroscopy because they do not enable near real-time operation [15–18]. In arthroscopic procedures, such as ACL reconstruction (knee) or FAI resection (hip), the objective of navigation is to control the position of the power tool to open the tunnel and carry resection, respectively. This requires vSLAM with (i) a reasonable frame rate (never below 15 fps), (ii) resilience to sudden occlusion, and (iii) high accuracy in pose estimation at every frame time instant, without relying on retrospective optimization such as the one carried in the situation of loop closing. Other deep learning-based vSLAM approaches operate in near real time [19, 20]. The problem with these methods is that they do not provide the camera poses or the reconstructed point cloud as an output, and further processing would be required to obtain such information.

Another family of SLAM methods includes the classical approaches. A classical SLAM pipeline typically consists of four main stages: point tracking, localization, mapping, and loop closing [21]. ORB-SLAM3 [21] is one of the state-of-the-art classical vSLAM methods, presenting top performance in real-world scenarios. However, its performance degrades significantly when applied to different endoscopic domains such as colonoscopy [8] and arthroscopy [9]. CudaSIFT-SLAM [8] and OneSLAM [9] have recently been proposed to overcome this limitation. CudaSIFT-SLAM redesigned ORB-SLAM for colonoscopy by (i) integrating the CudaSIFT implementation [22], an open-source GPU implementation of SIFT [23] for accelerated keypoint detection, and (ii) using GPU-accelerated Brute-Force (BF) matching for relocalization and place recognition, which can be advantageous in challenging scenarios with low or repetitive texture, as it supports more global reasoning beyond local texture similarity. Elvira et al. [8] demonstrated that CudaSIFT-SLAM produces more repeatable features, tracks a larger number of keypoints, and generates larger and more accurate maps than ORB-SLAM. Because of this, and by being purely monocular while providing camera poses and a 3D map of the scene in real time, this classical approach is a viable solution for performing VBSN in arthroscopy. OneSLAM [9], which employs deep learning for feature tracking, has shown superior performance compared to ORB-SLAM in arthroscopy and will be used as a baseline in this work.

### Arthroscopic dataset creation

As briefly mentioned in Sect. Introduction, the VBSN system employs an anatomy marker and a digitizing tool, both instrumented with visual markers, to enable surgical navigation. The anatomy marker with known visual patterns is a 3-mm metal cube with an attached threaded shaft that is screwed into bone. Although its implantation is invasive, it is placed in bone rather than cartilage or soft tissue; therefore, surgeons do not consider it to cause permanent anatomical damage due to the bone’s capacity for self-regeneration. The implantation procedure typically lasts less than 30 s and does not disrupt the standard clinical workflow. During navigation, these calibrated fiducial patterns are detected and tracked in the arthroscopic images, and their relative pose with respect to the camera is estimated at every frame time instant using 3D computer vision techniques [1]. Similarly, the 3D pose of the digitizing tool can be computed with respect to the camera by detecting its marker in each frame (refer to Fig. 1 in the Supplementary Material).

Over the past few years, our team has conducted several bioskills experiments using the VBSN system to obtain ground-truth registrations between the preoperative model and the anatomy marker. This process proceeds as follows: During cadaver experiments, several registration trials are performed. In each trial, a point cloud of the anatomical surface is acquired by moving the digitizing tool across the anatomy. A rigid transformation aligning the preoperative model to the acquired point cloud is then computed using the registration algorithm provided by [4]. Each resulting transformation is referred to as an intraoperative registration solution, representing a possible alignment between the acquired intraoperative 3D points and the preoperative model.

After collecting several sets of 3D points with the digitizing tool and registering them to the preoperative model, a tunnel location is planned and a guidewire is drilled along the planned trajectory. Its position and orientation are then acquired with respect to the anatomy marker using a special instrument, defining the captured tunnel. Following the intraoperative stage, each specimen undergoes a CT scan. The target anatomy and the guidewire are manually segmented, with the segmented guidewire defining the ground-truth tunnel. The line defined by the guidewire is intersected with the 3D model, yielding two extrema points: the entry point, located in the intercondylar region, and the exit point. These ground-truth points are projected onto the preoperative model by registering the pre- and post-operative 3D models, which is a straightforward geometric alignment given their large overlap.

For each intraoperative registration solution, the captured tunnel is projected onto the preoperative model. The errors between the captured and ground-truth tunnels, in terms of entry point displacement and tunnel direction difference, are used as quantitative metrics for assessing registration accuracy. Also, among all intraoperative registration solutions, the one minimizing both errors is defined as the ground-truth intraoperative registration. Both error metrics are used to evaluate the performance of the vSLAM-based registration in Section Contactless registration using Arthro-vSLAM+ and Contactless registration in VBSN.

## Specializing vSLAM for arthroscopy

Building upon the CudaSIFT-SLAM framework, which demonstrated promising results in endoscopic applications [8], we developed two variants specifically tailored for arthroscopic scenarios: Arthro-vSLAM and Arthro-vSLAM+, described in the following sections.

### Arthro-vSLAM: specializing CudaSIFT-SLAM for arthroscopic scenarios

Arthro-vSLAM extends the CudaSIFT-SLAM pipeline for arthroscopy by incorporating a deep learning-based semantic segmentation model to identify the target anatomy (bone and cartilage) that should be reconstructed to accomplish registration. Although vSLAM reconstructs 3D points from all visible intra-articular structures, the preoperative model used for navigation represents only bone and cartilage surfaces. As reported in [24], existing registration algorithms cannot handle the prohibitively high percentage of outliers that arise from reconstructing all visible contents in arthroscopic imagery (e.g., ligaments, fat tissue, and other bone structures). While correspondences on highly non-rigid regions can be eliminated through epipolar geometry verification, those in semi-rigid structures, such as ligaments, cannot be easily discarded. A semantic segmentation model enforces consistency between the intraoperative reconstruction and the preoperative model by excluding non-relevant structures; only 3D points from segmented bone and cartilage are used for registration. See Sect. Background and related work in Supplementary Material for details of the semantic segmentation model. Arthro-vSLAM uses the entire arthroscopic image for camera pose estimation (localization), while the segmentation mask is used for 3D structure estimation (mapping). Apart from the introduction of semantic filtering, Arthro-vSLAM follows the original CudaSIFT-SLAM pipeline [8] without further modification.

### Arthro-vSLAM+: combining Arthro-vSLAM with a fiducial marker attached to anatomy

Experiments in Sect. Can Arthro-vSLAM avoid the need of a fiducial marker attached to anatomy? show that Arthro-vSLAM still struggles to localize the camera and map structure inside the articular joint with sufficient accuracy and robustness to safely guide navigation throughout the procedure, particularly when precise control of power tools is required. This motivated the development of Arthro-vSLAM+, which requires a marker to be rigidly attached to the anatomy, as in the standard VBSN system, to accurately determine the camera relative pose while preserving the contactless 3D reconstruction capabilities of Arthro-vSLAM.

When the anatomy marker is within the camera’s FOV, Arthro-vSLAM+ receives the marker-based camera poses as input and reconstructs the 3D map. When the marker is not visible, either because it leaves the FOV or is occluded, the Arthro-vSLAM module is activated, allowing continuous 3D mapping of the scene. Map initialization follows the same strategy as in CudaSIFT-SLAM [8] and requires two frames with sufficient visual content and parallax. Maps are initialized only when the anatomy marker is visible, and camera pose optimization is disabled when poses are provided by the anatomy marker tracker. This hybrid approach enables the reconstruction of anatomical regions that would otherwise remain unseen if the anatomy marker had to remain visible at all times, thereby potentially expanding the area that can be reconstructed. By providing a larger area for reconstruction, Arthro-vSLAM+ opens the way for FAI surgery and other procedures, where it is difficult to both access the target anatomy with a touch probe and keep the anatomy marker in the FOV at all times.

Arthro-vSLAM+ offers two additional advantages. First, contrary to the control of power tools that cannot rely in retrospective optimization, the reconstruction of regions out of sight of the anatomy marker can benefit from retrospective bundle adjustment as a way to improve overall accuracy. Second, by taking advantage of the anatomy marker, which provides highly accurate camera pose estimations, feature matching in Arthro-vSLAM+ is performed in a guided manner. Specifically, instead of using BF matching to compare each descriptor in the first image with all descriptors in the second image, the matching process is constrained to features located within a fixed distance (10 pixels) from the corresponding epipolar line. When the anatomy marker is not visible and camera poses are instead estimated by SLAM, standard BF matching across all descriptors is used (see Sect. Specializing vSLAM for arthroscopy in Supplementary Materials).

## Experiments

This section describes the datasets and compares Arthro-vSLAM with the state-of-the-art, followed by the evaluation of Arthro-vSLAM and Arthro-vSLAM+ for camera pose estimation and 3D reconstruction using arthroscopic video footage acquired from 7 knee joints. Camera pose estimation is assessed by comparing the estimated poses with those obtained through the tracking of the anatomy marker, while the quality of the 3D reconstruction is evaluated by analyzing the registration performance.

**Table 1:** Description of the in-house arthroscopic sequences

Seq	A1	A2	A3	A4 (tool)	B1	B2	B3	B4	B5	B6
#F	1091	600	1844	1365	1091	600	1844	1260	1500	1500
VAM	95%	100%	70%	95%	61%	74%	69%	80%	59%	43%

# F denotes the number of frames, and VAM indicates the % of frames with Visible Anatomy Marker for which exist ground-truth camera poses obtained via anatomy marker tracking. Sequences A1 to A3 and B1 to B3 were acquired from the same specimens, whereas the remaining sequences correspond to different ones; in total there are three left and four right knees

**Fig. 2:**
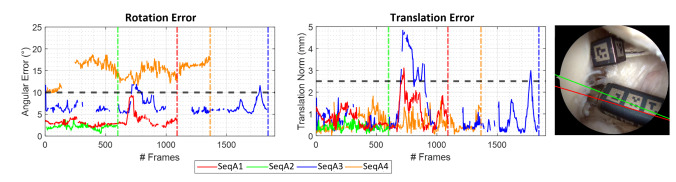
Camera pose estimation using the proposed SLAM approach. Seqs. A1–A4 from Table 1 were used, as they provide GT for the majority of frames. To illustrate the potential clinical impact of pose errors, deviations of 2.5 mm in translation or $10^{\circ }$ in rotation are considered. The right-side image shows the location of the femoral ACL tunnel that would be obtained by assuming a camera pose with errors of this magnitude (red line) compared to the ground-truth tunnel (green line) obtained by tracking the anatomy marker with submillimetric accuracy

### Datasets

The experiments conducted used 7 in-house arthroscopic sequences acquired from cadaveric ACL reconstruction procedures. In all sequences, the femur served as the target anatomy for registration. Each sequence was recorded at a resolution of $1080 \times 1920$ pixels and approximately 15 fps. Ground-truth camera poses were obtained by tracking the anatomy marker. Table 1 summarizes the arthroscopic sequences used throughout the experiments. For each sequence, it indicates the percentage of frames with available ground-truth camera poses obtained from anatomy marker tracking. Sequences Ai (i = 1...4) have been used to assess the ability of Arthro-vSLAM to recover camera pose (Sects. Comparison of Arthro-vSLAM with the state of the art and Can Arthro-vSLAM avoid the need of a fiducial marker attached to anatomy?). Sequence A4 occasionally has a surgical tool that occludes both the anatomy and the marker (see the rightmost image of Fig. 2). The sequences Bi (i = 1...6) include segments in which the anatomy marker was not visible. These are used in Sect. Contactless registration using Arthro-vSLAM+ to assess the usefulness of Arthro-vSLAM+ in extending the reconstructed area beyond the FOV of the anatomy marker.

**Table 2:** Root mean square of absolute trajectory errors (ATE), relative pose errors (RPE), and percentage of recovered cameras (coverage) obtained by the two methods

Arthroscopic sequences	Method	ATE (mm)	ATE (deg)	RPE (mm)	RPE (deg)	Coverage (%)
Sequences used in [9]	OneSLAM	0.93*	16.07*	**0**.49*	1.05*	100*
	Arthro-vSLAM	**0**.**77**	**11**.**35**	0.61	**0**.**84**	66
Seq.A1-Seq.A4 presented in Table 1	OneSLAM	3.02	19.90	1.00	3.36	25
	Arthro-vSLAM	**1**.**15**	**4**.**67**	**0**.**53**	**1**.**07**	**94**

Average results across all sequences are shown. Values with * were obtained from [9]. Best results in bold

### Comparison of Arthro-vSLAM with the state of the art

This section presents the comparative evaluation in camera pose estimation between the proposed Arthro-vSLAM framework and the state-of-the-art OneSLAM [9]. The authors of OneSLAM evaluated their algorithm against ORB-SLAM3 [21] on arthroscopic sequences of the knee joint with available ground-truth camera poses [11], reporting superior performance. To ensure a fair comparison, we used the same three sequences employed in their study and report the mean performance across Arthro-vSLAM and OneSLAM. In addition, we also compared Arthro-vSLAM and OneSLAM in in-house arthroscopic knee sequences (Sequences A1 to A4 in Table 1), which contain ground truth for the camera poses provided by tracking the anatomy marker. In these sequences, the anatomy marker was masked to avoid obtaining highly distinctive features that do not exist in real arthroscopic settings.

Table 2 reports the errors in camera pose estimation obtained with the Arthro-vSLAM and OneSLAM. In the sequences used in [9], OneSLAM achieves full coverage (100%) because it outputs camera poses for all frames, regardless of their tracking quality. In contrast, Arthro-vSLAM achieves lower coverage (66%) since it discards unreliable poses. For our in-house sequences (Sequences A1 to A4 in Table 1), the OneSLAM parameters were fine-tuned to reject poorly tracked frames. Arthro-vSLAM maintained higher accuracy and a more stable coverage, as its ORB-SLAM-based design inherently filters out low-confidence poses, ensuring that only reliable camera estimates contribute to the reconstruction. Overall, the experiments demonstrate that Arthro-vSLAM is the top performing method.

### Can Arthro-vSLAM avoid the need of a fiducial marker attached to anatomy?

This experiment uses Sequences A1 to A4 from Table 1, with the anatomy marker masked in all frames. During surgical navigation, accurate frame-by-frame camera pose estimation is essential, and the estimation must be resilient to the presence of surgical instruments. Based on extensive cadaver experiments and discussions with physicians, we consider camera pose errors above 2.5 mm and $10^\circ$ relative to the anatomy as thresholds, since it has been experimentally observed that exceeding these values can cause significant errors in navigation (see the rightmost image of Fig. 2), impacting the surgical outcome [25].

Post-optimization, such as loop closing, cannot be applied during real-time navigation, since the pose of the current frame must be available immediately to guide the surgical tools. These optimization steps typically refine past and current poses after processing future frames, which is incompatible with the strict real-time requirements of intraoperative navigation. For this reason, Fig. 2 presents camera pose estimates without these optimizations. Sequences A1 and A2 generally achieved accuracy levels acceptable for a surgical scenario, whereas Sequences A3 and A4 frequently exceeded the defined error thresholds. Sequence A4, which has the surgical tool, shows particularly high errors, reflecting the challenges introduced by the presence and movement of surgical instruments for markerless SLAM. These findings demonstrate that markerless SLAM remains challenging. Results obtained with post-optimization are included in Sect. Experiments of Supplementary Material for reference, but they do not represent the real intraoperative conditions considered here.

### Contactless registration using Arthro-vSLAM+

Building upon the previous experiments, this section evaluates whether vSLAM is beneficial for VBSN when the anatomy marker is visible during part of the sequence, and how this impacts registration accuracy. Arthro-vSLAM+ was tested on six sequences (Sequences B1–B6 in Table 1). For each sequence, Arthro-vSLAM+ was run 10 times, with each run reconstructing a surface point cloud and producing a single registration solution. Thus, each run corresponds to one intraoperative registration solution, resulting in 10 registration solutions per sequence. Registration is performed using the registration method proposed by [4].

**Fig. 3:**
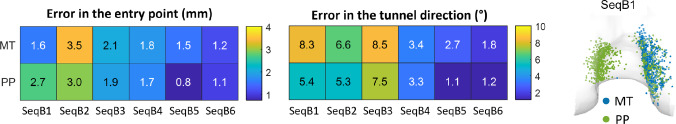
Registration results evaluated in terms of quality of ACL tunnel placement, as introduced in [24] and Sect. Arthroscopic dataset creation. Errors are median values computed for 10 runs of Arthro-vSLAM+. MT mode discards frames where the anatomy marker is not tracked. PP mode estimates the camera pose when the marker is not visible. The right side of the figure illustrates that PP mode provides more spatially distributed coverage of the anatomy (green points)

Figure 3 presents the registration results in terms of quality of ACL tunnel placement (refer to Sect. Arthroscopic dataset creation) obtained under two modes: (i) using only frames where the anatomy marker was successfully tracked (MT) and (ii) using all frames, where marker tracking was used whenever available and SLAM-based pose estimation was applied otherwise (PP). The results show that, although registration is feasible in the MT mode, the PP mode achieves an average reduction of $1.3^\circ$ in tunnel direction error. This improvement is expected, as the PP mode provides a denser and more spatially distributed reconstruction (see the right side of Fig. 3), facilitating more accurate registration, particularly in terms of rotational alignment.

### Contactless registration in VBSN

Arthro-vSLAM+ was tested live in a cadaveric knee, with contactless registration performed on the tibia for the first time. Three intraoperative registration solutions were performed on femur and tibia using Arthro-vSLAM+, each from a 1.5-min video sequence. For comparison, three additional registrations were conducted using the standard VBSN procedure with the digitizing tool. The registration method proposed by [4] runs in a separate thread and is triggered whenever the intraoperative reconstruction map is updated. Video 2 in Supplementary Material illustrates the contactless registration procedure with Arthro-vSLAM+.

**Fig. 4:**
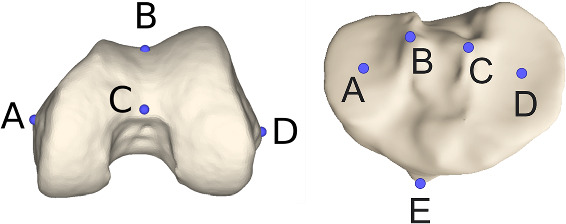
Control points (CP) on femur (left) and tibia (right)

Following the evaluation protocol described in [1, 26], four control points (CPs) on the femur and five on the tibia were used to assess registration precision (see Fig. 4). The CPs were defined on the corresponding preoperative 3D model and then transformed into the anatomy marker reference frame using the three registration solutions obtained per method. Consequently, for each method, each CP was mapped in three different ways. For each method, a centroid was computed for each CP after this mapping, and the RMS distance of each mapped CP to its centroid was calculated, with lower values indicating higher precision. Table 3 reports registration errors for both femoral and tibial experiments. In femur experiments, all errors were below 2 mm, with the digitizing probe achieving slightly lower errors than Arthro-vSLAM+. The “Agreement” column in Table 3 reports, for each CP and each anatomy, the RMS distances of the six mapped positions of that CP (three from the digitizing tool and three from Arthro-vSLAM+) to the centroid computed from these six positions. This metric quantifies the consistency between the two approaches. The RMS values for the femur in “Agreement” column are low, indicating that Arthro-vSLAM+ provides registration results comparable to the digitizing probe, with deviations at CPs B and D remaining below 2.5 mm. In tibia experiments, Arthro-vSLAM+ demonstrated higher precision than the digitizing probe at CPs A, D, and E, while agreeing closely with the probe at CPs B and C. The RMS distance in the “Agreement” metric reached 4 mm at CP E, indicating the largest discrepancy between the two registration approaches. Overall, these results suggest that Arthro-vSLAM+ achieves registration precision comparable to the digitizing probe.

**Table 3:** Registration error (mm) in ex vivo experiments for the control points of Fig. 4. **Probe** are the registration results obtain using standard VBSN using the digitizing probe, while **Arthro-vSLAM+ **shows the results for the proposed SLAM approach. The column **Agreement** reports the RMS distance between probe and Arthro-vSLAM+, providing a measure of consistency between the two methods

	Probe		Arthro-vSLAM+		Agreement	
CP	Femur	Tibia	Femur	Tibia	Femur	Tibia
A	0.75	1.34	1.30	1.00	0.87	2.49
B	1.53	0.57	1.85	0.79	2.40	1.17
C	0.73	0.50	0.79	0.61	0.76	1.42
D	1.86	0.91	1.98	0.48	2.39	2.64
E	–	2.33	–	1.04	–	4.02

All values are in millimeters (mm)

**Table 4:** Reconstructed surface quality for femur and tibia using the standard probe and Arthro-vSLAM+

Anatomy	Probe		Arthro-vSLAM+	
	RMSE (mm)	Coverage (%)	RMSE (mm)	Coverage (%)
**Femur**	0.73 ± 0.20	12.65 ± 0.23	2.84 ± 0.07	18.46 ± 0.15
**Tibia**	1.67 ± 1.38	18.96 ± 1.06	1.42 ± 0.37	19.81 ± 1.01

Surface RMSE, denoted as **RMSE** (mm, mean ± standard deviation), and surface coverage, denoted as **Coverage** (%, mean ± standard deviation), are reported for each anatomy, computed from three registrations per method

The quality of registration was further evaluated in terms of ACL tunnel placement, as described in Sects. Arthroscopic dataset creation and Contactless registration using Arthro-vSLAM+. For the femur, the standard VBSN using the digitizing tool yielded a mean error of $(3.6 \pm 0.7)$ mm and $(2.4 \pm 1.1)^{\circ }$, whereas Arthro-vSLAM+ achieved $(3.4 \pm 1.1)$ mm and $(1.7 \pm 1.3)^{\circ }$. For the tibia, the standard VBSN obtained $(3.6 \pm 0.1)$ mm and $(4.3 \pm 1.0)^{\circ }$, while Arthro-vSLAM+ reached $(2.2 \pm 0.8)$ mm and $(5.4 \pm 1.4)^{\circ }$. These results demonstrate that Arthro-vSLAM+ provides comparable tunnel placement accuracy than the standard method.

Additionally, two metrics were used to assess the quality of surface reconstruction: (i) the surface RMSE between reconstructed points and the preoperative model and (ii) the surface coverage of reconstructed points over the region of interest (ROI). Using the three registration solutions per anatomy and per method, all reconstructed points were aligned with the corresponding preoperative model, and the RMSE of each reconstructed point to the registered model was computed. Surface coverage quantifies the fraction of the anatomical surface ROI that is reconstructed with sufficient accuracy. Specifically, coverage is defined as the ratio between the number of ROI model points lying within a 1-mm radius of the reconstructed points and the total number of model points in the ROI. Results are reported in Table 4. For the tibia, both RMSE and surface coverage are comparable between the two methods. This is consistent with the tunnel placement accuracy results, where similar performance is observed for the two approaches. For the femur, the standard VBSN procedure using the digitizing probe achieves a substantially lower RMSE than Arthro-vSLAM+. However, Arthro-vSLAM+ provides a larger surface coverage, meaning that a broader portion of the region of interest is reconstructed. This increased spatial coverage appears to contribute to the slightly better tunnel placement accuracy observed on the femur compared to the standard probe.

These results demonstrate that Arthro-vSLAM+ attains registration accuracy comparable to that of the standard VBSN using the digitizing tool, while eliminating the need for direct instrument contact. This represents a significant advantage, as manual digitization can be time-consuming, technically challenging due to the probe’s limited range of motion, and poses a risk of iatrogenic damage to delicate anatomical structures such as cartilage. In addition, Arthro-vSLAM+ runs in real time at approximately 20 fps (see Sect. Specializing vSLAM for arthroscopy of the Supplementary Material).

## Conclusion

This paper demonstrates, for the first time, the feasibility of real-time contactless registration within the VBSN framework for arthroscopy using a SLAM-based approach. The proposed Arthro-vSLAM+ can operate with camera poses from either anatomy marker tracking or SLAM-based localization when the marker is not visible, enabling 3D reconstruction of anatomical regions despite temporary occlusions. Additionally, this work presents the first successful deployment of contactless registration in tibial arthroscopy, highlighting the broader applicability and potential of SLAM-based approaches in regions that are difficult to access with a digitizing probe.

## Supplementary Information

Below is the link to the electronic supplementary material.

Supplementary file 1 (mp4 30463 KB)

Supplementary file 2 (mp4 59128 KB)

## References

[1] Raposo C, Sousa C, Ribeiro L, Melo R, Barreto JP, Oliveira J, Marques P, Fonseca F (2018) Video-based computer aided arthroscopy for patient specific reconstruction of the anterior cruciate ligament. In: MICCAI. Springer-Verlag, Berlin, Heidelberg, pp 125–133, 10.1007/978-3-030-00937-3_15

[2] Figueroa F, Figueroa D, Guiloff R, Putnis S, Fritsch B, Itriago M (2023) Navigation in anterior cruciate ligament reconstruction: state of the art. J ISAKOS 8(1):47–53 36179977 10.1016/j.jisako.2022.09.001

[3] Raposo C, Barreto JP (2017) Using 2 point+normal sets for fast registration of point clouds with small overlap. In: ICRA, pp 5652–5658, 10.1109/ICRA.2017.7989664

[4] Raposo C, Barreto JP (2018) 3D Registration of curves and surfaces using local differential information. In: CVPR, pp 9300–9308

[5] Kim Y, Lee BH, Mekuria K, Cho H, Park S, Wang JH, Lee D (2017) Registration accuracy enhancement of a surgical navigation system for anterior cruciate ligament reconstruction: a phantom and cadaveric study. Knee 24(2):329–339. 10.1016/j.knee.2016.12.007 28189409 10.1016/j.knee.2016.12.007

[6] Ren H, Kazanzides P (2012) Investigation of Attitude Tracking Using an Integrated Inertial and Magnetic Navigation System for Hand-Held Surgical Instruments. IEEE/ASME Trans Mechatron 17(2):210–217. 10.1109/TMECH.2010.2095504

[7] Mahmoud N, Cirauqui I, Hostettler A, Doignon C, Soler L, Marescaux J, Montiel JMM (2017) ORBSLAM-based endoscope tracking and 3D reconstruction. In: Computer-Assisted and Robotic Endoscopy. Springer International Publishing, pp 72–83

[8] Elvira R, Tardós JD, Montiel JMM (2024) CudaSIFT-SLAM: multiple-map visual SLAM for full procedure mapping in real human endoscopy. arXiv:2405.16932

[9] Teufel T, Shu H, Soberanis-Mukul RD, Mangulabnan JE, Sahu M, Vedula SS, Ishii M, Hager G, Taylor RH, Unberath M (2024) OneSLAM to map them all: a generalized approach to SLAM for monocular endoscopic imaging based on tracking any point. Int J CARS 19(7):1259–1266. 10.1007/s11548-024-03171-6 10.1007/s11548-024-03171-6 38775904

[10] Marmol A, Corke P, Peynot T (2018) ArthroSLAM: multi-sensor robust visual localization for minimally invasive orthopedic surgery. In: IROS, pp 3882–3889, 10.1109/IROS.2018.8593501

[11] Marmol A, Banach A, Peynot T (2019) Dense-ArthroSLAM: dense intra-articular 3-D reconstruction with robust localization prior for arthroscopy. IEEE Robot Autom Lett 4(2):918–925. 10.1109/LRA.2019.2892199

[12] Wang Y, Long Y, Fan SH, Dou Q (2022) Neural rendering for stereo 3D reconstruction of deformable tissues in robotic surgery. In: MICCAI, Cham, pp 431–441

[13] Wang K, Yang C, Wang Y, Li S, Wang Y, Dou Q, Yang X, Shen W (2024) EndoGSLAM: real-time dense reconstruction and tracking in endoscopic surgeries using gaussian splatting. In: MICCAI. Springer Nature Switzerland

[14] Zha R, Cheng X, Li H, Harandi M, Ge Z (2023) EndoSurf: neural surface reconstruction of deformable tissues with stereo endoscope videos. In: MICCAI, pp 13–23

[15] Liu X, Li Z, Ishii M, Hager GD, Taylor RH, Unberath M (2022) SAGE: SLAM with appearance and geometry prior for endoscopy. In: ICRA. IEEE Press, pp 5587–5593, 10.1109/ICRA46639.2022.9812257 10.1109/icra46639.2022.9812257 PMC10018746 36937551

[16] Wang S, Leroy V, Cabon Y, Chidlovskii B, Revaud J (2024) DUSt3R: geometric 3D vision made easy. In: CVPR, pp 20697–20709, 10.1109/CVPR52733.2024.01956

[17] Zhu Z, Peng S, Larsson V, Cui Z, Oswald MR, Geiger A, Pollefeys M (2024) NICER-SLAM: neural implicit scene encoding for RGB SLAM. In: 2024 international conference on 3D vision (3DV), pp 42–52, 10.1109/3DV62453.2024.00096

[18] Leroy V, Cabon Y, Revaud J (2024) Grounding image matching in 3D with MASt3R. In: ECCV. Springer-Verlag, Berlin, Heidelberg, p 71–91, 10.1007/978-3-031-73220-1_5

[19] Rosinol A, Leonard JJ, Carlone L (2023) NeRF-SLAM: real-time dense monocular SLAM with neural radiance fields. In: IROS, pp 3437–3444, 10.1109/IROS55552.2023.10341922

[20] Liu Y, Dong S, Wang S, Yang Y, Fan Q, Chen B (2024) SLAM3R: real-time dense scene reconstruction from monocular RGB videos. arXiv:2412.09401

[21] Campos C, Elvira R, Rodríguez JJG, M. Montiel JM, D. Tardós J, (2021) ORB-SLAM3: An accurate open-source library for visual, visual-inertial, and multimap SLAM. IEEE Trans Rob 37(6):1874–1890. 10.1109/TRO.2021.3075644

[22] Björkman M, Bergström N, Kragic D (2014) Detecting, segmenting and tracking unknown objects using multi-label MRF inference. CVIU 118:111–127

[23] Lowe DG (2004) Distinctive image features from scale-invariant Keypoints. Int J Comput Vision 60(2):91–110. 10.1023/B:VISI.0000029664.99615.94

[24] Baptista T, Raposo C, Marques M, Antunes M, Barreto JP (2024) Keypoint matching for instrument-free 3D registration in video-based surgical navigation. In: MICCAI. Springer Nature Switzerland, Cham, pp 339–348, 10.1007/978-3-031-72089-5_32

[25] Samitier G, Marcano AI, Alentorn-Geli E, Cugat R, Farmer KW, Moser MW (2015) Failure of anterior cruciate ligament reconstruction. Arch Bone Jt Surg 3(4):220–240 26550585 PMC4628627

[26] Baptista T, Marques M, Raposo C, Ribeiro L, Antunes M, Barreto JP (2024) Structured light for touchless 3D registration in video-based surgical navigation. Int J CARS 19(7):1429–1437. 10.1007/s11548-024-03180-5 10.1007/s11548-024-03180-5 PMC11230986 38816650

